# Changes in the immigrant Russian-speaking family language policy during the war in Ukraine

**DOI:** 10.3389/fpsyg.2024.1385420

**Published:** 2024-05-31

**Authors:** Ekaterina Protassova, Maria Yelenevskaya

**Affiliations:** ^1^Department of Languages, Faculty of Arts, University of Helsinki, Helsinki, Finland; ^2^Technion – Israel Institute of Technology, Haifa, Israel

**Keywords:** family language policy, war in Ukraine, flexibility in language use, changing linguistic identity, Russian as an international language

## Abstract

**Introduction:**

The Russia’s full-scale invasion of Ukraine had a significant impact on the Russian-speaking identity, reformulating existing linguistic and cultural boundaries and shaping Russian speakers’ self-perception and vision of the world. We focus on families with children who are trying to balance their inner and outer life in order to stabilize the positive environment of the upbringing process and analyze how adults explain to the children the need to learn the Russian, Ukrainian and other languages. This can shed light on the challenges and strategies employed in alleviating prejudiced attitudes against immigrants’ languages and cultures which can cause alienation from the roots in the one-and-a-half and second generation. The research questions were: How has the war altered language policies in families with different ethnic backgrounds? What changes in home language use strategies do parents propose?

**Material and methods:**

Material drawn from numerous posts of Facebook discussions have been analyzed with the help of thematic analysis.

**Results and discussion:**

We observe that in many multilingual families with Ukrainian roots the war has led to a greater emphasis on the Ukrainian language use as an attempt to reinforce ties to their cultural heritage and express support for the country. Russian is increasingly viewed as the language of the aggressor; moreover, Russian culture is devalued and rejected. Some families have become more open to new language learning, especially the languages of their new environment, and try to identify themselves as multicultural and multiethnic personalities. On the other hand, in those families where parents work or study in the fields in which Russian is widely used, adults prioritize the development of Russian language skills in their children in order to improve their future educational and professional opportunities. We explore attitudes and challenges faced by parents in Russian-speaking families, as they demonstrate the complexities of identity formation and language transmission while making salient the interplay between parents’ personal experiences, their aspirations for their children’s cultural identity, and the pressures of integration into the local society.

## 1 Introduction

Historically, the Ukrainian and Russian languages coexisted in Ukraine since the late 16^th^ century, and the policy of Russification began in the 18th century (Hosking, 1997; Kumeda, 2024). In the Soviet times there were periods when Ukrainian was promoted^1^ and when it was suppressed, but it had the status of an official language of the country (Bilaniuk, 2018; Danylenko and Naienko, 2019; Shvedova, 2021). Russian has been widely spoken in many regions, particularly in the east and south (*cf.* Zeller and Sitchinava, 2020). Bilingualism was widespread in different domains and particularly well developed among the urban population (e.g., Pavlenko, 2012; Kanishcheva et al., 2023). However, the current conflict has intensified linguistic tensions between Ukrainian and Russian speakers, with both sides using language as a means of political and cultural expression (Arel and Ruble, 2006; Hentschel et al., 2014; Knoblock, 2019). The Russian government is using the conflict as an opportunity to promote its own values, portraying the war as a fight for the soul of the nation. The myth of a “Great Russian” identity positions Russia as the protector of Russian speakers around the world. This identity is a construct based on the idea of a shared linguistic, cultural, and historical heritage and has been used to justify Russia’s intervention in Ukraine (Fedotova, 2024; Protassova and Yelenevskaya, 2024a).

Among Ukrainian residents and citizens, besides ethnic Russians there are Albanians, Armenians, Crimean Tatars, Greeks, Hungarians, Jews, Karaims, Slovaks, and others. Some of them identify themselves as Ukrainian or Russian or have affinity with other ethnic or cultural groups in which they see their family roots (Melnyk and Csernicsko, 2010; Myshlovska, 2018). Considering a large number of mixed marriages, many have hybrid identities. Today, in the face of aggression people of diverse ethnic backgrounds rooted in Ukraine have begun to identify more strongly with Ukraine. Nevertheless, the war’s impact on individual identities is fluid and nuanced, and some of its aspects may contradict each other. Thus, rejecting Russian altogether for “patriotic” reasons may be impractical, because for decades it was widely used as a *lingua franca*^2^ by these communities. Romaniuk (2014) explores trends in the development of native language education and finds that in Ukraine, the focus is on state language policies and national consciousness, while in the Western diaspora (USA and Canada), external factors (language policy in the country of residence and pressure to assimilate) as well as internal factors (national consciousness, the need to integrate, and education in the Ukrainian as the native language) play crucial roles.

Efforts to promote the use of standard Ukrainian are going on in various spheres, but *Surzhik*, a mixed Ukrainian-Russian variety, continues to have a presence in everyday speech of many Ukrainians in some regions. This mixed sociolect, or a blend of Ukrainian and Russian languages is predominantly spoken in areas where both Ukrainian and Russian-speaking populations coexist, and speakers combine elements of both languages in their speech. The use of *Surzhik* has been a topic of debate and controversy in Ukraine, as some view it as a degradation of the purity of Ukrainian, while others see it as a natural consequence of cultural and linguistic interactions in the region (Friedman, 2010, 2023; Masenko, 2019; Hentschel, 2024).

Kulyk (2017) discusses how political representation of Russian-speaking citizens in Ukraine impacted language-related policies and politics. Russian-speaking citizens wielded influence by electing representatives at various government levels. For instance, Leonid Kuchma’s victory in 1994, backed by Russian-speaking voters, was influenced by his promise to enhance the status of the Russian language. Although this support did not guarantee unrestricted Russian-language use, the sway of Russian-friendly politicians affected legislation, preventing extreme Ukrainianization measures during different presidencies. Eventually, Viktor Yanukovych’s victory at the Presidential elections in 2010, led to a 2012 language law elevating the legal status of Russian and thus alienating Ukrainian nationalists. The mobilization of voters in eastern and southern regions by emphasizing ties to Russia and the Russian language caused discord among Russian-speaking Ukrainians. Political parties aiming to achieve a linguistic balance which would reflect Ukraine’s diversity sought to avoid exclusive representation of any language or region. This complex interplay affected the country’s political landscape, language laws and regional divisions, and consequently, eroded the national unity.

According to Puleri (2020), in the years following Euromaidan, a debate in Ukrainian Studies emerged regarding the role of the Russian language and culture in Ukraine. Participants, and among them historians, political scientists and writers, discussed whether being a Russian speaker molds a political identity which potentially impacts loyalty to Ukraine. Some emphasized the blurred line dividing cultural and political Russophones^3^, noting the role of the Russian language as a common means of communication between various ethnic groups. Others, supporting this view, highlighted the absence of a distinct Russian-speaking group with a unified political identity due to geographical and societal complexities. A probable future of “two cultures-two languages” in Ukraine was also suggested. The main ideas highlighted in the discussion revolved around the status of the Russian-language literature in Ukraine and its recognition as Ukrainian literature. While there exists a rich Ukrainian Russian-language literary scene, it is not officially acknowledged as Ukrainian literature due to the absence of official bilingualism which was suspected to be a factor that could lead to further division of society. Some Russophone authors have shifted towards writing in Ukrainian, motivated by the desire for recognition in the Ukrainian literary landscape, particularly in the context of reduced ties with the Russian market amid the ongoing conflict. This shift reflected a global trend where Ukrainian and Russian cultures intersect and cross-fertilize each other, suggesting a hybrid cultural dynamic molded by local contexts. The dynamic which evolved in the Ukrainian Russophone literature after 2014, when the Crimea was annexed, might have set a precedent for similar cultural changes in the post-Soviet regions, but the new reality after 2022 was different (see also Littell, 2024).

This stance is mirrored in the poem written and rewritten by Boris Khersonsky (Ukrainian poet currently living in the USA) between 26.03.20 and 9.11.23 (*Я розмовляю російською с жінкой та наодинці,* Ukrainian for “When alone, I speak Russian to my wife”). Affected by the events, he changed both the content and the wording of the poem more than once. First, he wrote predominantly in Russian, but this changed after the war started. The latest version presents a complex mix of emotions and identities that evolve. In his poetry Khersonsky touches on such themes as language, cultural identity, religious conflicts, political power, and a sense of rejection (*cf.* Uffelmann, 2022). He discusses his use of Russian language with his wife, his attempts to learn Ukrainian, and the inner conflict between being a baptized Christian (although probably rejected by other Christians) and trying to maintain a Jewish identity (although rejected by other Jews). He feels he is an outcast and expresses a desire to return to Odessa, yet he fears to be rejected or physically harmed by its residents.

Odessa, a city with many Russian-speaking inhabitants, has seen a shift towards an increased use of Ukrainian since the onset of the war. Some residents have actively transitioned to speaking Ukrainian more frequently, which resulted in higher fluency. Others, however, express reluctance, stating they may need to learn or improve their Ukrainian in order to use it in formal settings, but they are unlikely to use it regularly in their daily lives. This change in language use reflects evolving sentiments and practical adaptations of Odessa’s residents amidst the conflict^4^.

Many Russian Jewish identities have roots in Ukraine, as Ukraine has a long and complex history of Jewish settlement and cultural exchange with local inhabitants. The American singer and composer Regina Spektor (Nelson, 2023) identifies herself as a Russian-speaking or Russian Jew, questioning whether this identity stems from the historical antisemitism haunting Jews in the Soviet Union and Russia. She believes that Jews in Russia never truly felt entirely Russian; rather, they considered themselves Russian Jews due to societal perceptions that separated them from all the others. She reflects on her family’s diverse roots, with grandparents being originally from Ukraine and Belarus, parents born in Ukraine, but ultimately, they all “come from Moscow.” As immigrants early on they realized the constructed nature of identities. This is vividly seen given her family’s varied origins: one grandmother, whose maiden name is Berlin, was from Zhitomir near Kyiv, the other one, with a Polish-sounding surname, was from Belarus. This diverse heritage makes them feel like seeds that have been everywhere. Ms. Spektor acknowledges the significance of nationalism, especially during times like war, when it becomes crucial for people to feel a sense of belonging and safety.

According to testimonies of our interviewees (Protassova and Yelenevskaya, 2024b), before the war, Ukrainian communities in such countries as the Czech Republic, the United Kingdom, and Italy, were integrated into the cultural life of the Russian-speaking communities, even if Ukrainians outnumbered Russians. Despite the domestic policy of Ukrainization and intentions to promote the Ukrainian language among the diasporans, there were almost no efforts to organize Ukrainian schools abroad, supplying them with teaching materials and curricula fitting the needs of heritage speakers^5^. Therefore, language maintenance among members of the one-and-a half and second-generation immigrants was mostly limited to everyday conversations in the family, which seldom promises proficiency. On the other hand, Russian schools appeared in many European cities. Their teachers accumulated and exchanged experience and gradually gained reputation for preparing their students for using the language both in informal and formal settings. Since Russian is a global language, speaking it means to be part of a large transnational intercultural community^6^, and Ukrainian speakers were often among members of Russophone groups. Today, however, many of them have stopped using Russian as a sign of protest, and switched to Ukrainian on Facebook, YouTube, Telegram, and when answering questions of various surveys. Some immigrant Russophone families search for Ukrainian ancestors. Immigrant Russian-speaking families, particularly those with roots in Ukraine, reconsidered their language policies. For example, parents have made a conscious effort to follow the Ukrainian history courses and to reorient their children to Ukrainian. This has become a topic of societal discussion: What are the motives behind it? Is it deep-felt solidarity with Ukraine? Do they want to be treated as Ukrainians who have sympathies of the people in the West, or do they seek exemption from collective responsibility for Russia’s actions?

In this article, we try to answer two research questions:

- How has the war affected language policies within families with diverse ethnic backgrounds?
- What approaches can parents adopt to adjust their strategies regarding the language used at home?

## 2 Materials and methods

Material for the study was drawn primarily from Facebook (FB) discussions in the groups uniting parents, primarily mothers, wishing to exchange experience of child rearing away from the home country. The posts and discussions that followed were gathered for a week in May 2023. The posts and comments were contributed by approximately one hundred participants from at least 25 countries spanning all continents. These virtual communities have become very popular because many families face dilemmas of how to bring up multilingual children. Most of these groups are open for viewing to any FB member. In order to express your opinion, you have to sign up, but as a rule, administrators grant permission in case you accept the rules (usually requiring mutual respect, banning abusive language, and sometimes prohibiting advertising). Even though some of the discussants anonymize themselves, we made sure that they would be unrecognizable by omitting their demographic data. We do not give names of the groups in which discussions were carried out. We change or omit the name of the country in which discussants currently reside, change or omit the towns of the participants origin, gender of the children, and where it is not relevant to the gist of the discussion their age. The outcomes are contingent upon the analytical focus, methodological choices, and ethical approach toward participants involved in the activities and contexts under study.

We were interested in the discussion threads addressing questions related to early development, bilingualism, and speech therapy assistance for diasporic families. Participants, share their experiences of raising children in culturally diverse environments. We believe that this type of opinion exchange is most natural and, therefore, reliable for conducting qualitative analysis. In addition, we compared our findings with evidence from other sources (e.g., Leikin et al., 2014; Tsimpli, 2014; Armon-Lotem and Meir, 2019).

Thus, our project uses textual data from a variety of sources and employs thematic analysis. This is a method for identifying, analyzing, and reporting patterns (themes) within data collected for a project (Braun and Clarke, 2006). That is to say, the goal is to find patterns across an (entire) data set, rather than within individual data items. An important feature of the thematic analysis is that it is not bound to any pre-existing theoretical framework, and so it can be used within different theoretical frameworks, which contributes to the flexibility of the method. When dealing with new phenomena data coding starts with search for themes in the collected texts. As bigger chunks of material are accumulated and studied, the formulation of the themes may change (Vaismoradi et al., 2013; Nowell et al., 2017; Braun and Clarke, 2019). Moreover, theoretical assumptions that the researchers had when launching the project, may also be modified under the influence of the empirical material collected. When analyzing our data we kept in mind that thematic analysis incorporates both manifest and latent aspects of the phenomena studied. It means that the analysis of latent content of data is an inseparable part of the manifest analysis. This made us pay special attention to the explicit and implicit meanings expressed by the participants. We find thematic analysis fitting the material we study, because it helps in-depth understanding of the phenomena, it safeguards researchers from overemphasizing or neglecting key themes in discourse and it does not lead to the disruption of the participants’ communication. The salient themes that we singled out during the analytical work appear as headings in section 4.

Social media platforms are increasingly being utilized as standard tools for parents to share and exchange their educational experiences and practices (Goodyear et al., 2014). They serve as natural focus groups, following the initiative of the parents themselves. In fact, educators and caregivers benefit from participating in these forums since they have an opportunity to look at bilingual child-rearing problems from a different perspective. There is cross-fertilization of competencies, and professionals utilize these forums to launch constructive discussions (Kelly and Antonio, 2016; Macià and García, 2018; Robson, 2018). Encouraging more parents and teachers to express their views can facilitate discussions. As a rule, administrators create the atmosphere of openness, tolerance, and constructive criticism. It is crucial to empirically examine parental online interactions as potentially valuable new forms of transnational discussions of expats, refugees, or other migrants, despite their unpredictability compared to established professional interviews.

## 3 Home language use and identity brokering

Family functioning and narrative identity are interconnected, as family experiences and dynamics provide the foundation for children’s sense of self and identity development. Education, language, and identity are intricately linked aspects of human development and social interaction (Nortier, 2018). Language plays a crucial role in education, as it is the primary medium through which knowledge and information are conveyed (Rothbart, 2011). The language used in education can impact learning outcomes, especially for students whose first language is different from the language of instruction. A lack of alignment between language and education can hinder educational achievement and contribute to identity-related challenges (Figueras and Masella, 2013). Socio-economic factors often influence educational opportunities. The type of education individuals receive can impact their future opportunities and socio-economic mobility, which, in turn, can influence their sense of identity and belonging in society (Mastrotheodoros et al., 2021). When individuals learn a second language in a new cultural context, they often engage in a process of acculturation, where they adopt elements of the host culture. This leads to inevitable changes in identity as individuals adapt to new social norms, values, and ways of communication, although they might not be aware of it themselves (Paris, 2011).

Positive family interactions, open communication, supportive environments, and adaptive responses to challenges—all contribute to the formation of a coherent and non-contradictory identity. Supportive family responses to the challenges of migration fosters resilience and adaptive identity development in children and adolescents (Hoyt and Pasupathi, 2009; Cierpka, 2014). Multiliteracy in education expands the traditional concept of literacy to include various modes of communication and expression. It equips children with skills to navigate the digital age, fosters creativity and collaboration, and influences their identity development by exposing them to diverse perspectives and communication styles. Multiliteracy supports the formation of dynamic and adaptable identities capable of coping with complexities of a rapidly changing world (Ibrahim, 2016).

Identity may also differ across generations within a family. First-generation migrants usually identify more strongly with their country of origin, while second-generation migrants gradually come to be more connected to the host country. In countries with high levels of xenophobia or anti-immigrant sentiment, and in case of conflicts between the ancestral home-country and the host country migrants, including youngsters, often develop a stronger sense of solidarity with their fellow-migrants and may identify more strongly with their country of origin. Such “reactive ethnicity” often evolves in the face of perceived threats, persecution, and discrimination. This is one of the modes of ethnic identity formation, pointing to the role of a hostile context of the immediate environment or political upheavals and wars in the home country which may strengthen ethnicity rather than erode it (Rumbaut, 2005). Individuals may experience multiple identities simultaneously; as time goes on, they may acquire new ones or at least partially shed those they brought from the home country, so identities constantly evolve under the influence of events in a person’s life, e.g., diasporic, borderline, and transnational identities emerge (*cf.* Abreu Fernandes, 2019; Karpava et al., 2021; Protassova et al., 2021).

Children are sensitive to changes in their environment. Let us consider two case studies. The first is from our interviewees’ pool. Oksana, a five-year old girl from a Russian-speaking family in Mariupol, became a refugee to Germany, escaping bombing together with her aunt in 2022. She made friends with her peer Nina, born in Germany of a Russian mother and German father. The children began playing together, but Nina, who had not spoken Russian before, although her comprehension was very good for her age, was upset that her new playmate did not understand German. First, she summoned her mother to act as an interpreter, but since the mother was too busy, she had to cope herself and to the delight of her Russian-speaking family members finally started speaking Russian. Hoping that their refugee life would end soon, Oksana’s aunt tried to prepare the child for school in Ukraine. Some of the exercises were in Russian, but others were in Ukrainian. Two years have passed. Both children go to school now. They continue speaking Russian to each other, only occasionally switching over to German. However, Oksana speaks German to Nina’s two-year-old sister Anna.

Another example of a child’s language shift^7^ due to changing circumstances is Misha, who was 6 years and 9 months old during the interview with his parents. He was born and spent his infancy in Kyiv, while his mother and grandmother are from Donetsk, and great-grandmother lives in the Crimea. As acknowledged by his parents, he spontaneously transitioned to speaking Ukrainian. This shift occurred roughly 3 months into family’s evacuation to Lviv in western Ukraine. At home, parents conversed mostly in Russian, but while strolling outdoors, in Ukrainian, exposing Misha to the Ukrainian language environment. One day, something seemed to click for the child. Now he enjoys speaking Ukrainian and identifies himself as a Ukrainian. More than a year after this language shift, he started ‘relearning’ some Russian phrases, albeit mainly for amusement or to playfully tease his parents. At the same time, he often corrects his parents’ and grandmother’s *Surzhyk* enjoying his role of their mentor (Yakovlev, 2023).

The language dynamics and cultural preferences among Ukrainian and Russian speakers, particularly those living in Finland, reflect the usage of Russian and Ukrainian within families, as well as the desire to maintain Ukrainian traditions which differ from Finnish customs. In a 2022 survey (Protassova, 2024), among speakers of Russian with different home languages (140 participants), there were five Ukrainian families. One respondent mentioned that Russian takes 80% of the time in her family, and Ukrainian and Finnish 10% each; in another family, Russian was employed 60% of the time, and Ukrainian 40%; in the third, Ukrainian was used for 90% of the time, and Russian 10%; in the fourth, Ukrainian was used for 20% of the time, Russian for 30%, and Finnish for 50%. One family did not specify the division of languages. All participants wanted to maintain Ukrainian in the family communication. One of them wrote: “We should maintain traditions, maybe not all of them, but the main ones, which we have in Ukraine. We must observe them differently from Finnish traditions, for example, in the celebration of Christmas and Easter.” Another survey showed that Ukrainians who immigrated to Finland wanted to stay in the country, whereas Russian families could imagine that their children would live somewhere else (Koskimies and Gusatinsky, 2022). Even these few individuals demonstrate different levels of preference for Russian and Ukrainian; moreover, the survey highlights Ukrainian families’ desire to preserve their cultural heritage and traditions but also become fully integrated in Finland, while some Russian families might be more open to the idea of their children living outside Finland.

## 4 Results

There are many discussions about the use of languages in migrant families and in discussion groups of Russian-speaking diasporans. This section presents several discussion topics that drew attention of numerous participants of the FB-groups we monitored. We reproduce the main contents of discussions conducted by Russian- and Ukrainian-speaking mothers. The quoted and reproduced posts enable us to see which topics appeared on the parents’ agenda as a result of the Russia’s full-scale invasion of Ukraine.^8^

### 4.1 What is the right pace for changing the language for a young child

The discussion opens with PL’s request for advice on how to introduce a new language, or rather replace it by another one: *My daughter is less than two-and-a-half years old. I speak Russian with her, and my husband speaks French; our environment is English-speaking, and my husband and I speak English to each other. Our daughter speaks in short sentences (“Mommy, let us go there,” “It’s a big car,” “Mama come here,”* etc.*) in all languages in the same way. How do I replace the Russian language by Ukrainian; Is it better to switch abruptly and completely, or somehow gradually, and if gradually, then how? You know, I tried it once and she was a little confused, maybe I need to warn her about the language change so that she understands why she does not understand me well now*; *Unfortunately, I do not have any Ukrainian-speaking people nearby yet. Little by little I started switching on cartoons in Ukrainian for her to see. I have already found some channels on Telegram with Ukrainian audio fairy tales; I will try them.*

Commentators’ recommendations vary. Some think that as long as the author’s daughter is so small and has a small vocabulary in Russian, she should go right away to Ukrainian. They are convinced that the child’s age is favorable for this, and no problems will arise. This means that the mother should replace all content immediately and completely: books, nursery rhymes, music, animation films, in order to “restart.” They also think it would be useful to “overload” the child as much as possible. Words are understandable in context, yet talking is another matter. If she does not understand something, parents should explain it in English or whatever language is convenient. YX: *Just start talking. Anything she does not understand, reformulate it and show it clearly; at first, sometimes you’ll duplicate some words in Russian, but very soon this will not be necessary. Just at this age, 3 years ago, our dad switched completely from Ukrainian to Russian (for educational purposes—the main language of our family is Ukrainian), and after 2 months my son began speaking Russian without any problem. Now, he is completely trilingual (Ukrainian is his main native language, Russian is weaker but also fluent, and Italian is the language of the environment). Good luck to you!* Some supporters of the immediate switch express reservations. They caution that truly vulnerable children, or those who have already demonstrated sensitivity to languages may deserve a special approach. ZA hopes: *Look at the child’s reaction. Perhaps everything will go very smoothly, completely unnoticed*.

Some other commentators favor slow replacement in standard situations, such as dinner time, when the names of different foods are acquired. A step-by-step process is viewed as easier for a child. One can start with 15 min a day in Ukrainian, gradually increasing the amount of the new language use. When understanding of Ukrainian improves, a gradual switch to Ukrainian would be unproblematic. Familiar books, the child’s favorites, are worth reading in Ukrainian, and the mother should assemble a children’s library in Ukrainian. Cartoons and audiobooks can be found in the public domain. Famous cartoons can be watched in different languages. Audiobooks are just a lifesaver for KM, because her seven-year-old listens in two languages instead of binge-watching cartoons. Some audiobooks are based on cartoons.

Participants endorse parents’ switch to Ukrainian which should encourage the children and help them catch up. They report how they shifted together when the child was six or eight. They add that they also used Ukrainian when talking with friends, for example. Dozens of families, according to posts, do this. One author goes so far as to suggest that conscientious parents should stop dreaming in Russian, apparently unaware that the physiological and psychological mechanisms of dream sleep cannot be controlled by ideology^9^. Depending on the readiness of the parents, the family can shift to Ukrainian completely. But if they do not plan to make full transition, in a couple of years they can send their child to a Saturday Ukrainian school and watch and read only Ukrainian content. In one discussion thread a role model for some participants is SP who presently resides in Switzerland. As a child, she effortlessly transitioned from speaking Russian to Ukrainian. Initially, she swiftly adapted, yet later she realized that Russian was not to be given up, due to limited opportunities to speak Ukrainian, apart from interactions with relatives in Ukraine. At that time, she lived in Latvia.

TT: *It depends on how ready you are to move. If you yourself can switch in one fell swoop, then, switch completely and at once. It was like that for us. I could not switch right away, I forgot a lot myself so, I made an effort to speak Ukrainian at least 1 h a day. And I gradually increased the time and this is how I transferred many words from passive to active* [knowledge]*. Now I speak Ukrainian all day plus I’ve started reading books in Ukrainian, plus cartoons. My child was 3 years old at that time. Additionally, we were in a new country, so the child had to learn two new languages virtually simultaneously. At first, I simply translated separate words into Russian for him. Then I explained new words (as I thought) in Ukrainian but in different words* [apparently, she means that she explained the meaning of the new words the way she understood it herself]. *And only after 8 months it gave results. The child began speaking to me in Ukrainian. So, try, and if you have a persistent desire* [for your child to speak Ukrainian]*, then do not give up if there is no result right away.*

In sum, commentators suggest either an immediate, complete switch by immersing the child in Ukrainian content across various media, emphasizing contextual understanding and gradual language exposure, or a slower, step-by-step approach focusing on integrating Ukrainian into daily activities like mealtime, reading, and media consumption, depending on the child’s sensitivity to language and individual reactions.

### 4.2 The similarities between Russian and Ukrainian: do they facilitate the family language shift?

Acquiring closely genetically related languages may not necessarily be easy due to subtle differences in vocabulary, grammar, and pronunciation that can present challenges for learners. Additionally, many seemingly identical words may radically differ in meaning. A group of discussants think that it is easy for a Russian-speaking child to understand Ukrainian than start with other languages because Russian and Ukrainian are very similar. They claim to understand 100 percent of what is said in Ukrainian, using food names as an example of equivalence between the words forming the basic vocabulary. They believe the child will not even notice the switch. But one participant objects sarcastically:

BV: *Yes, very similar. In Ukrainian,* breakfast *is* сніданок*, in Russian, it is* завтрак; to speak *is* разговаривать *in Russian and* розмовляти *in Ukrainian,* to look *is* смотреть *in Russian and* дивитися *in Ukrainian. 90% of words are not the same. Your suggestion is wrong. I am now dealing with this issue since my child is six and we are switching to the Ukrainian language. It is precisely because the words are not similar that it is difficult to switch. Our Russian neighbors understand 10% of what is said in Ukrainian. In addition, neither Russian nor Ukrainian are the languages of the environment. He also speaks to dad in a different language, which is not the language of the environment.*

Some participants are aware that everyday situations are the least problematic, since in addition to the actual linguistic means, extralinguistic ones, i.e., body language and the situational context are helpful, but at a more advanced level it is more difficult to understand all the subtleties. Indeed, a lot of words are virtually the same, which allows one to watch Ukrainian television. Ukrainian folk songs are pleasant to sing, and children memorize them quickly.

Discussants admit that although both languages are Eastern Slavic, on a deeper level, difficulties emerge. All Slavic languages are mutually understandable to some extent (Slovak and Polish are mentioned); the listener can grasp the idea but cannot communicate. Nevertheless, there are children who speak three Slavic languages almost without confounding them. The phonetics are completely different, e.g., in Ukrainian and Russian sibilants are pronounced differently. Participants distinguish between learners who are Slavic and non-Slavic native speakers. For the native speakers of non-Slavic languages who know Russian very well, understanding Ukrainian is hardly possible. For those Russians who have heard some Ukrainian as children, almost everything is understandable. Yet, children will master both *palyanitsya* and *spіdnitsya* (a traditional loaf of bread and a skirt—both words serve as shibboleths in Ukrainian). Syntactic constructions are similar, and children are fast to accumulate vocabulary if the content interests them. Some commentators observe that Ukrainians are bilinguals themselves and do not realize how different the languages are. It is necessary to carefully monitor whether the child understands new words.

Additional concern for the parents is the quality and authenticity of the language. Many dialects are spoken in Ukraine, and not everyone has mastery of literary Ukrainian. So, a question often arises what sort of Ukrainian will the children learn if their only interlocutors are family members. Another worry is emotions of the child: would it be stressful if all of a sudden mother started addressing him/her in a “foreign” language? So, one of the suggestions is for the parents to detach the decision from ideology and try to conduct self-assessment of their own Ukrainian language proficiency, aiming beyond everyday conversation. Would they be able to explain in Ukrainian such complex concepts as DNA, differentiate between bacteria and viruses, or elucidate the natural water cycle? The parents fear that the result of their efforts might be their children’s hybrid *Surzhik*, rather than fluency in either “good” Russian or “good” Ukrainian. Yet, they point to the growing availability of the Ukrainian content and believe that its relevance will increase in the coming years. The perceived familiarity with the language from childhood might not match the actual present proficiency level, especially if speaking Russian predominated for a long period. Therefore, an objective evaluation of one’s language skills and the availability of Ukrainian resources become crucial in making an informed decision. Discussants realize that only a minority of parents would be able to explain scientific matters to their children in a didactically adequate way even in their first language, in particular if it was not the language of instruction in their own school. It does not often happen that children learn about science, technology and society from their mothers and grandmothers, those very people who are usually most involved in child rearing. Yet, even if you do not have all the themes of the school curriculum at your fingertips in your native language, it is not a reason not to sing lullabies in it.

All in all, while some discussants argue that Russian-speaking children can easily understand Ukrainian due to similarities between the languages, particularly in basic vocabulary like food names, others highlight significant differences in vocabulary and phonetics, suggesting that the transition may not be as seamless, especially considering contextual and extralinguistic factors. Clearly, everyday situations may be less problematic, as extralinguistic cues facilitate comprehension. Whatever the disagreements, all participants acknowledge the complexities inherent in mastering both languages, with some noting differences in phonetics and syntactic constructions, and others emphasizing the importance of monitoring the child’s comprehension and progress.

### 4.3 Organization of language acquisition process in multilingual homes

The process of language acquisition and how to organize it best concerns most of the participants of the forums:

KO: *It’s not just about understanding, the child must start speaking. I’m not talking about the necessity to mull over familiar situations for a long time; you can gently switch to Ukrainian in a couple of weeks. Your son will quickly start to speak. These languages have the same grammar, the same phonetics. All that remains is vocabulary (and even there a lot is in common) and pronunciation.*

Parents experienced in bringing up trilinguals and quadrilinguals emphasize the importance of consistency and perseverance. They believe that when you introduce a new language to a child, you must speak it all the time. They also see the difference between additive and subtractive multilingualism^10^ and suggest introducing a new language rather than replacing the one/s the child already speaks. Notably, the ability to translate and interpret are seen as a special one. In fact, many immigrant parents who have not achieved proficiency in the language/s of the host society often make the mistake of trying to use their adolescent children in these capacities unable to understand that these are professional skills.

*First, I introduced common words, what everything around is called, and then used them in sentences. In this way you will succeed. I did not often translate but showed everything in action, so that the child could understand. When we learn the first language, we do not get translation, we simply speak and accept it in practice, and with a second language, we take the words for granted without interfering with the first language and without confusing them. Now, my child speaks four languages and can translate himself. Although we tried not to translate at all. At first, when I started speaking non-Russian in sentences, my daughter resisted and tried to force me to speak her first language, saying that what I’d said was incorrect, and repeated or answered in her first language, Russian. Everything has its time; rollbacks are also the norm in the beginning. At the start, it happened that I reproduced almost entire dialogues in books as a translator when I read. Later I stopped when I* *realized that she had understood, and I just spoke or switched to books in another language.* One can also read the same books in different languages.

A family shares their experience of introducing a third language to their five-year-old, because this is the wish of the Belarusian father. They integrate Belarusian into everyday interactions by subtly introducing easy phrases or naming familiar objects in Belarusian, ensuring the child’s comfort and gradually increasing familiarity with the language. Positive and festive events are shared in Belarusian, while they avoid discussions involving unpleasant topics or reprimanding the child in the new language. They incorporate Belarusian into various media such as cartoons, books, songs, and bedtime stories, occasionally making exceptions and switching over to Russian. Additionally, the family practices a “word of the day” routine, learning two Belarusian words daily from tear-off calendars, engaging both the child and the parents in the learning process. They also play flashcard games and ask riddles, making language learning enjoyable and interactive for the entire family. Moreover, adults, belonging to different ethnicities, emphasize cultural elements such as embroidered shirts, pottery, national flags, and stories about their native countries, fostering a sense of pride in their national identity. Through language and cultural appreciation, they aim to instill a stronger sense of belonging and loyalty to their respective heritages.

Some discussants warn their virtual interlocutors that although they understand the parents’ desire to introduce a new language, they think that if it means mastering four languages it is too much. But in one of such cases the child apparently has aptitude for languages, given the three-language-environment and three-word sentences the child could produce at the age of 2.6. Yet, if the mother switches to Ukrainian, Russian may be abandoned. One of the participants, DK speaks about the combination of languages in her family: the father is a Portuguese speaker, the environment is German, and the mother’s languages are Russian and Ukrainian. She admits that *the process is not easy and it’s good if you start early. The key thing is that you want it, which means everything will work out. It’s difficult for us because my daughter already has a huge vocabulary in Russian and reads Russian fluently*. *We started reading very simple books in Ukrainian because her comprehension is poor for her age. The words are very different. And unfortunately, Ukrainian is the only language my daughter speaks with an accent. For example, she pronounces* litáki *and not* litakí [aircraft]. Some mothers give examples of Ukrainian words amusing and puzzling their children, such as *gudziki* or *shkarpetki* [buttons, socks].

Another case is discussed by a parent raising 7-year-old twins who attend a Ukrainian school on Saturdays. The parent communicates with the children in a 50–50 mix of Ukrainian and Russian. Acknowledging that transitioning completely to Ukrainian would be easier if the children were younger, the parent emphasizes the importance of exposing them solely to Ukrainian language content in cartoons and songs. Despite the complexity of managing four languages, the twins have achieved notable progress in Ukrainian proficiency within a year. The parent aims to eventually establish Ukrainian as the primary language, supported by the grandparents who reside with the family. The grandparents converse in Ukrainian to each other, providing additional reinforcement, while the parent communicates with them exclusively in Ukrainian, which also seems to aid the children’s language acquisition process.

We see that parents and forum participants discuss strategies for language acquisition, emphasizing the importance of consistency and immersion, and sharing experiences of integrating new languages into daily interactions through gradual exposure, positive reinforcement, and interactive learning activities. Challenges such as language accent and vocabulary differences are addressed, and the parents are aware of complexities of their task and the necessity to introduce language in context and foster cultural appreciation alongside linguistic development. Despite concerns about managing multiple languages, success stories highlight the benefits of early exposure and consistent reinforcement, with children demonstrating significant progress in language proficiency over time.

### 4.4 Languages learned in homes abroad: how authentic are they?

Children are flexible and they get used to new situations quickly. It takes up to 6 months to switch over to a new language in a kindergarten. Progress in language acquisition depends on many factors, ranging from the child’s abilities and interests, to the atmosphere in the family and attitudes to multilingualism in society. Some parents observe that stability in the family is to a large extent maintained through the use of the same home language. Nevertheless, some children even if they come from the same family and are brought up according to the same principles, behave differently, and those who spoke their first language more fluently than their siblings might later give it up completely, while the others maintain it.

TE: *I have four children. The eldest is 15 years old, and he came to the U.S.A. at six. Before that he had spoken both Russian and Ukrainian fluently. The two youngest ones were born in the US, and they spoke only Russian (we do not count English). When the war began, my cousin’s wife and daughter came to stay with us. The daughter speaks only Ukrainian, she does not know any Russian. All of us immediately switched to Ukrainian. Our youngest son protested at first, he was 5 years old, but I explained to him why we wanted to switch to Ukrainian, and he ceased being indignant. Now he speaks exclusively Ukrainian at home (by exclusively, I mean he does not use Russian at all, only English and Ukrainian). I think the presence of another child speaking good Ukrainian helped him a lot. My daughter understands Ukrainian and knows how to say a few words, but she cannot speak it fluently. She is only ten, and her Russian is very bad, she mostly speaks English.*

The situation has changed dramatically in the last years, and there is a lot of Ukrainian content available. An early start forms a basis for future autonomous learning. The parents realize that their children will decide themselves what language/s to speak when they grow up. They overwhelmingly agree that learning good English is pressing. The sequence of didactic actions remains the same: working on understanding, turning receptive vocabulary into productive speech, increasing immersion into the language. To effectively initiate and sustain language learning, establishing friendships with Ukrainian speakers is highly beneficial. Drawing from personal experience, participants mention that children become strongly engaged during playtime, particularly when interacting with friends who do not speak their home language. This interaction significantly enhances the child’s involvement in the language-learning process. A good solution is to invite a grandmother to live with the family. If the parents’ first language is Russian, it might be reasonable to delegate the function of speaking Ukrainian to somebody else like a nanny or a tutor, while the grandmother can perform a different function:

YL: *My daughter is Russian, she is married to a Ukrainian man, and they have two amazing twins almost 5 years old. At the initiative of my daughter, they completely switched over to Ukrainian at home, which I understand and accept completely. But, as an exception, I am allowed to teach children Russian. Children love this language, and I am happy that I can be a guide to Russian culture for my grandchildren. Yet, I have to strictly filter what to read to them, since Russian children’s literature has discredited itself too much.* In fact, many parents complain that some of the latest children’s books are badly written, and do not inspire good feelings. So, participants in the discussion recommend reading classic fairy tales or translations of modern foreign literature because the realities are easier to understand for the children living away from Russia.

According to VE, it took her family a year to make a transition from Russian to Ukrainian. Although in the beginning her daughter was “freaking out” in her protests, patience and work helped achieve desired results. When you get up in the morning, never forget to speak Ukrainian only, remove all Russian content. Then she switches over to Ukrainian. Children will force other relatives to speak на рідній мові [native language]. Головне повірити в те що це можливо і все починається з батьківського прикладу. Успіхів. [Most important is to believe that it’s possible and everything starts with parents’ example. Good luck.] AK adds in Ukrainian: Можливо з часом заговоримо виключно українською [Perhaps, with time, we will speak exclusively Ukrainian].

PC: *In my opinion, the key lies in fostering mental flexibility. Across* var*ious languages, it is evident that not only do phrases vary in sound, but their meanings also differ subtly. Language, in essence, intertwines with distinct thinking patterns, and having exposure to multiple languages broadens one’s mental horizons. It expands the scope of understanding the world and other people’s perspectives.*

To conclude this section, we can confirm that children are adaptable and can quickly adjust to new language situations, with language acquisition typically taking up to 6 months in a kindergarten environment, influenced by factors such as the child’s abilities, family atmosphere, and societal attitudes towards multilingualism. While stability in the family is often maintained through consistent use of the home language, individual children may exhibit varying language behaviors. Frequent interaction with peers and immersive language environments can significantly enhance language learning engagement resulting in higher proficiency.

### 4.5 What strategies work?

As children grow up their motivation to learn languages often changes influenced by their communication outside home and in particular by interactions with their peers. Some children are unwilling to switch over to Ukrainian and excessive pressure may lead to active opposition to the adults’ efforts. Therefore, members of the group discuss arguments that can support the language shift and justify it for the children. Many people had Russian as their first and dominant language, although their Ukrainian was quite good as well. The situation has changed, and now, they prefer to speak Ukrainian.

A mother of four children calls on the discussants not to be afraid and speak to the children only in Ukrainian. Her own family is in Tunisia where the language situation us complicated: at school children are exposed to French, English, written and Tunisian Arabic, plus they are exposed to Russian. Her youngest child had not spoken any of these languages except Russian before starting pre-school education at the age of three. Now, he speaks all these languages without an accent and is the best student in his class and second-best chess player in his age group in Tunisia.

EF: *I myself switched to Ukrainian at the everyday level a long time ago. I speak Ukrainian with my family and friends, but my son does not want to. He whines, “Mom, speak Russian.” My husband speaks Hungarian. At school, there is English and French. There are many languages* [in the child’s environment]*, I understand it is not easy. But Ukrainian was among his first languages. The question is how can we instill love for a language...?*

For IS, the challenge lies in resource management within the family, especially considering the presence of two languages added to the languages of the environment. Juggling three languages can be challenging but handling four demands skills and a significant amount of effort and patience, and not everyone is willing to commit him/herself to it.

One participant cites a frequently reiterated wisdom: the more languages the child is exposed to, the easier it will be for him/her later.

EV: *There is no problem “replacing” the language here. But I would not replace Russian with Ukrainian, even though I have roots both there and there* [in Russia and Ukraine]*. My main roots though are Jewish, but my grandparents did not pass the language on to us* [apparently, she refers to Yiddish]*. I really regret it now. So, think about it. The war will end*. OR immediately objects: *When the war stops, Russian will not be needed anymore*. Reminding participants that their discussion is conducted in that very “redundant” language JT retorts: *Then you yourself stop reading and writing anything in this language*. In fact, it is ironic that discussions in which some participants agitate for giving up the Russian language are conducted in Russian, thus testifying that Russian still remains a *lingua franca* for people whose origin is in the post-Soviet states.

The parents observe that a small child forgets the language which he/she stops hearing very quickly. For example, a boy had a Spanish-speaking nanny and spoke her language, which disappeared as soon as the nanny left. Another family had Italian in the environment, including kindergarten until the child’s 2.4, but when the family moved to Germany, the boy lost it completely, although he had already understood everything and could answer questions.

CK refers to those residing in Ukraine: *I would alternate between a day in Russian and a day in Ukrainian. I have relatives in the Kyiv region who speak and use both languages fluently (parents and children). Why reset and cancel something when everything still boils down to the economy?*

With a small child, who first acquires knowledge about everyday things, one should follow familiar routes – home, a road to the garden, a favorite playground, etc. On the way, adults can name people and objects they see in Ukrainian, whereas for more abstract things, one should use books. Parents should be very careful and introduce vocabulary in portions and thematic groups, such as furniture, crockery, or parts of the body through massage. They can start with rituals that are important and necessary for developing healthy habits. Participants share experiences as to how to turn learning into an interesting game. One can make a corner with books and toys, where they can speak and play only in Ukrainian. Some clothes might have the role of a magic wand: when one puts on a certain T-shirt, they speak only Ukrainian. One can buy new toys that only want to speak Ukrainian while the parent and the child are teaching them.

Children’s language acquisition and motivation can be influenced by their environment and with some children reluctant to switch to Ukrainian without understanding the rationale behind the transition, prompting discussions among parents on how to instill love for the language and manage multilingual resources within the family. In many discussions concerns arise regarding language authenticity, emotional stress, and attainment of high proficiency levels, with suggestions for parents to assess their own language skills and provide diverse learning experiences through games, rituals, and thematic activities to foster language development and engagement.

### 4.6 Is there wisdom in abandoning a language for ideological reasons?

The world situation is not favorable for the Russian language. Participants are sympathetic to the plight of Ukrainians and their reluctance to speak Russian. The Russian government is doing everything to ensure that the Russian language is becoming *non grata*. They speak prison slang themselves. Those who preferred Russian over Ukrainian changed their preferences after 22 February 2022. Some developed fear of speaking Russian with their children in the street even if they had not lived in Russia since 2010. This has never happened before. Some participants admit that it becomes more and more challenging for them to motivate themselves to continue speaking Russian. Especially in the first months after the start of aggression, people did not want to continue speaking Russian publicly with their children. Yet, there are others who feel that so much was taken away from them—their identity, good memories of their past, effortless contacts with their loved ones—that they are not prepared to surrender and allow politics to take away their language. Russian does not belong to “them,” it is the immigrants’ native language and the language of communication with their children.

Overall, language functions primarily as a means of communication rather than a vessel for specific values. According to linguists, it is inappropriate to label a language as inherently good or bad. Everything is possible only if the environment is safe and no inadequate adults are in the vicinity. Russian speakers from Ukraine (e.g., children from Kharkiv) are not aggressive; on the contrary, they invite those children who already live abroad and speak Russian to play and not to be afraid. Arriving in safe places in Europe, some Ukrainian refugees did not want to speak to Russian-language helpers in respective countries. Nevertheless, they had to accept volunteers’ support if they wanted efficient help from Job Centers and Social Services. There are many more diasporans and members of host countries proficient in Russian than those who know Ukrainian, although the situation is changing rapidly.

RB: *To be honest, in the 1.5 years I have never encountered inadequacy although I interact with refugees every day. And my eldest child was generally very happy that he could use the Russian language in communicating with* [refugee] *children and even act as an interpreter.*

In many places where refugees are received the atmosphere is tense. Some adults from Ukraine and Poland and even “local” Ukrainians, who migrated in the 1990s, broke up with their Russian friends, simply because they are Russians. Participants believe that Ukrainians have a moral right to demonstrate aggression towards the Russian language. Such attitudes, HJ comments, can be found anywhere today demonstrated by people of different nationalities. In the Netherlands, some Dutch-speaking people would reprimand immigrants, “speak the local language, since you live here.” One may manage *to shift the conversation to the topic of multilingualism and how great it is when you can give children several languages. Well, in the context of the current situation, you just need to sensibly assess the situation and avoid a conflict if, God forbid, it arises.* For RI, nothing can justify aggression towards a child for speaking Russian, in particular if we consider, that he is bilingual and only one of his parents is an ethnic Russian.

Many discussants in the groups we observed find switching to Ukrainian unacceptable for them personally, but they regret that some people in their environment are passive-aggressive, and refuse to acknowledge that Ukrainians are a nation, that the Soviet Union ceased to exist already a long time ago and that each country which once was part of it is now an independent state.

PL: *This is the language my mother spoke to me; it is native to me. I do not care what anyone thinks. According to this principle, Germans should all stop speaking German. But I understand you: at least once a week they ask me why we are studying Russian with our children instead of Ukrainian*. PN gives a detailed answer to this question. During World War II, ethnic Germans residing in the USA were ashamed and scared to speak their language, abstaining from teaching it to their offspring. Their descendants think that abandonment of their native language was a misguided response and are sorry that their parents and grandparents failed to teach them their heritage language. The narrator often pondered on this, distinguishing language from the ideology of Nazism. However, the outbreak of the war in Ukraine caused a profound shift in the PN’s perspective, prompting significant personal growth. Despite Ukrainian heritage, PN had limited formal education in Ukrainian, studying it only twice a week for an hour. Russian served as the family’s primary language throughout her life. The onset of the war compelled the narrator to reflect. Through her experiences and subsequent contemplation, PN gained empathy for the quandary faced by those who grappled with preserving their language and cultural identity in challenging times. After a long inner struggle, PN arrived at the conclusion that one’s heritage language is not to be abandoned.

EG: *My child’s knowledge of a second language (in our case, Russian) is much more important for me than what other people think. And if you do not speak the language, then it’s obvious that it will disappear, especially among children.*

A psychologist and speech therapist, the moderator of one of one of the groups discussing early bilingual education, often gives recommendations to the parents. They can be summarized as follows: *When considering teaching a language to your child, it is crucial to first determine your purpose and motivation behind this decision. Assess all available resources—both material and non-material, including time and energy necessary for effective language instruction. Ensure the availability of sufficient learning materials for the chosen language and start accumulating them to support the learning process. Reflect on the decision to eliminate one language in favor of another. Evaluate whether it is truly necessary or beneficial to limit the development of two languages. Consider whether it holds value to encourage a child to abandon a language she already speaks. Recognize that the techniques and methods employed for fostering language development should remain consistent regardless of the language being taught. Ultimately, the language your children will speak is a decision and responsibility that lies with you. It is important to deliberate thoughtfully and consider the implications of your choices for your child’s linguistic development and cultural identity.*

Participants express concerns about the diminishing status of the Russian language and empathize with Ukrainians’ reluctance to speak it, highlighting tensions and shifting attitudes towards language use amidst geopolitical conflicts. While some defend the preservation of their native language as a matter of personal identity and heritage, others grapple with societal pressures and advocate for the importance of bilingualism and multilingualism in fostering cultural appreciation and communication.

## 5 Discussion

The impact of social media platforms as increasingly ubiquitous tools for all human activities, including professional practices, necessitates further exploration, although our analysis of the data was limited to a qualitative approach. Analysis of the material we collected confirms growing awareness of the parents of methods of multilingual education. The dilemmas of how to bring up multilinguals in the most efficient way draws parents together. On the one hand, few are ready not to rely on their own intuition; on the other hand, today, lay people are much more open to recommendations of their peers and professionals. FB communities uniting parents, including the ones we have monitored for this project, enhance parents’ confidence, help them build social networks and share experiences and pedagogical knowledge. Together with the professionals participating in the observed groups, their members support each other in creating a positive learning environment and working out role models of behavior (*cf.* Cohen and Anders, 2020).

Users’ judgments of language functions, forms and speakers’ behaviors may be idealized or biased. Some participants’ decisions to speak Ukrainian and reject Russian, the language which served as their home language and the first language of their children, is caused by their attitude to the war waged by Russia against Ukraine. Making this decision parents do not always realize the complications it involves. The first one is that at the moment there are few offline opportunities to delegate the task of teaching Ukrainian in the diaspora to professionals, which means that the responsibility lies entirely with the family. Secondly, some of the parents have only limited proficiency in Ukrainian, so their efforts may be ineffective. Of course, there are modern means to promote the language and to improve one’s command of it studying autonomously, yet not all people are concentrated enough to use them systematically. Notably, their self-assessment as regards proficiency in Ukrainian may be wrong, since at least some of them are only familiar with the vernacular and have never been exposed to literate and academic Ukrainian. Thirdly, the children who already speak Russian sometimes protest and are unwilling to switch over to a new language. The situation for the children in the diaspora is particularly stressful if they speak different languages with different members of the family and in the kindergarten or school. If the war continues long, bringing more devastation to Ukraine, one can hypothesize that the number of people rejecting the Russian language may increase. The more people accept the language shift, the more it will be seen as normal. Support of the media, religious organizations and creation of educational institutions capable of teaching Ukrainian to young heritage speakers will further reinforce the process of the shift.

Rejection of the Russian language and replacing it by Ukrainian should be qualified as subtractive bilingualism. At the same time, surveys and interviews, as well as FB discussions, clearly indicate that the majority of the parents in the diaspora value heteroglossia and theoretically wish their children to be active multilinguals in at least two or three languages. In fact, this requires more than the knowledge of vocabulary and grammar skills, but also cultural knowledge, such as politeness norms, etiquette, values, and behavioral norms of the ethno-cultural group sharing the same language.

In sum, the decision regarding language usage with children is highly personal, and families often have to grapple with various dilemmas. A sudden and complete removal of one language, like Russian, might cause confusion or resistance, leading to emotional distress. It is advisable to double the linguistic exposure by spending more time with Ukrainian-speaking grandparents, considering the involvement of a Ukrainian-speaking nanny, or enrolling in a Ukrainian school. Every family crafts a unique approach tailored to their circumstances. Although abrupt transitions should be approached cautiously to avoid causing distress, there are well tested methods applicable to any language pair. Employing Ukrainian textbooks and educational materials aids in language development.

The war has influenced language policies in families with diverse ethnic backgrounds by prompting shifts in linguistic practices and priorities. Many shifted to Ukrainian, promptly or consequently. Some families adopted a more inclusive approach, embracing multiple languages as a means of preserving cultural heritage and promoting unity amidst adversity. Others prioritized the dominant language for practical reasons such as ensuring communication with external support networks or managing new educational opportunities for their children.

For effective and enjoyable learning experiences, seeking guidance from professionals who specialize in teaching Ukrainian to children, such as an online tutor which involves games increasing the child’ motivation and encouraging interactions can be highly beneficial. Sometimes, parents who initially acquired languages themselves could not foresee the impact it would have on their children’s linguistic development. All in all, the choice of language is an individual and complex decision that shapes a child’s linguistic journey and cultural identity.

## 6 Conclusion

Many people in Ukraine have mixed heritage, and may identify themselves both with Ukrainians and Russians, or with other ethnic or cultural groups (Maksimovtsova, 2020; Shevchuk-Kliuzheva, 2020; Braha, 2021; Sokolova, 2022). Similarly, many people with roots in Ukraine who identify themselves as Russian speakers have not changed their identity or allegiances as a result of the conflict. At the same time, people of diverse ethnic backgrounds rooted in Ukraine have begun to identify more strongly with Ukrainian unity in the face of aggression (*cf.* Boiko and Vintoniv, 2023; Plokhy, 2023). In many bilingual families, the war has prompted a greater emphasis on the Ukrainian language use as an attempt to reinforce ties to their cultural heritage and express support for the country (*cf.* Nedashkivska, 2018; Masan et al., 2022). Todorova (2023) claims that while foreign mentoring programs show promising results in addressing various challenges faced by displaced individuals, domestic initiatives lack the breadth of application seen abroad, necessitating the development of conflict-sensitive emotional mentoring tailored to the needs of internally displaced Ukrainian families (*cf.* Altynbekova, 2024). In fact, the impact of the war on individual identities is difficult to predict because there is a multitude of factors involved, such as one’s family story, domicile (whether one lives or lived in the area close to fighting), resilience and resistance to stress, and many others. Above all, it is whether a person has lost his or her loved one/s on the battlefield or as a result of shooting and bombing.

The conversations among parents bringing up bi-and trilingual children revolve around transitioning from speaking Russian to speaking Ukrainian. Many parents seek advice on whether to switch languages abruptly or gradually. Opinions differ; some suggest an immediate shift, citing linguistic similarities between the languages, while others advise a gradual transition, recommending exposure to Ukrainian through daily routines, books, and media. Concerns about language differences, vocabulary, and pronunciation challenges are discussed. It is acknowledged that a complete switch might be initially difficult, emphasizing the importance of supporting the child’s understanding and speaking ability in the new language. Clearly, this is not a matter of 1 day or 1 month, as one person puts it. In Ukraine, those who have never spoken Ukrainian started speaking it not out of fear, but out of contempt: they do not want to speak the same language as the aggressor.

To adjust their language strategies at home, parents can consider several approaches: they can encourage the use of both the dominant language and the native language(s) spoken within the family while fostering a sense of identity and connection to cultural roots. They can recognize the evolving needs of the family and adjust language policies accordingly because of the changing circumstances brought about by the war. They can find ample opportunities for language exposure through activities such as reading, storytelling, and cultural celebrations which support language development in children. They also engage in dialogue with family members about language preferences and concerns possibly facilitating understanding and collaboration in maintaining linguistic traditions. They can try to access resources and support networks, such as community organizations or online forums, that offer guidance and encouragement in overcoming language-related challenges during times of conflict (*cf*. Fedyuk and Kindler, 2016 Seals, 2019).

New identities in Russian and Ukrainian migrant families can take on many different forms and may be shaped by a variety of factors such as cultural background, personal experiences, and the social and political context of the host country. Many individuals develop hybrid identities that incorporate elements of both their cultural background and their experiences in the host country. Migrants adapt to the norms and values of the host country in order to fit in and succeed, which leads to changes in identity, as individuals may adopt new cultural practices, language, or values that differ from those of their country of origin. On the other hand, some individuals resist assimilation and maintain a strong connection to their cultural roots. This manifests in various ways, such as maintaining close ties to the community, speaking their native language at home, observing traditions or participating in cultural events organized for the community and members of the host society.

## Data availability statement

Publicly available datasets were analyzed in this study. This data can be found at: https://www.facebook.com/groups/126340711067392.

## Ethics statement

Ethical approval was not required for the study involving human data in accordance with the local legislation and institutional requirements. Written informed consent was not required, for either participation in the study or for the publication of potentially/indirectly identifying information, in accordance with the local legislation and institutional requirements. The social media data was accessed and analyzed in accordance with the platform's terms of use and all relevant institutional/national regulations.

## Author contributions

EP: Writing – review & editing, Writing – original draft, Visualization, Validation, Supervision, Software, Resources, Project administration, Methodology, Investigation, Funding acquisition, Formal analysis, Data curation, Conceptualization. MY: Writing – review & editing, Writing – original draft, Visualization, Validation, Supervision, Software, Resources, Project administration, Methodology, Investigation, Funding acquisition, Formal Analysis, Data curation, Conceptualization.
